# Assessing the outcome of Strengthening Laboratory Management Towards Accreditation (SLMTA) on laboratory quality management system in city government of Addis Ababa, Ethiopia

**DOI:** 10.11604/pamj.2015.20.314.5375

**Published:** 2015-03-31

**Authors:** Abay Sisay, Tedla Mindaye, Abrham Tesfaye, Eyob Abera, Adino Desale

**Affiliations:** 1 Addis Ababa Health Research and Laboratory, Addis Ababa, Ethiopia; 2 Departement of medical Laboratory science, College of Health Science, Addis Ababa University, Addis Ababa, Ethiopia; 3 Addis Ababa University, College of Natural Science, Addis Ababa, Ethiopia; 4 Quality Africa Network/GIZ, Addis Ababa, Ethiopia; 5 Ethiopian Public Health Institute, Addis Ababa, Ethiopia

**Keywords:** SLMTA, WHO-AFRO, mentorship, accreditation

## Abstract

**Introduction:**

Strengthening Laboratory Management Toward Accreditation (SLMTA) is a competency-based management training programme designed to bring about immediate and measurable laboratory improvement. The aim of this study is to assess the outcome of SLMTA on laboratory quality management system in Addis Ababa, Ethiopia.

**Methods:**

The study used an Institutional based cross sectional study design that employed a secondary and primary data collection approach on the participated institution of medical laboratory in SLMTA. The study was conducted in Addis Ababa city government and the data was collected from February ‘April 2014 and data was entered in to EPI-data version 3.1 and was analyzed by SPSS version 20.

**Results:**

The assessment finding indicate that there was a significant improvement in average scores (141.4; range of 65-196, 95%CI =86.275-115.5, p = 0.000) at final with 3 laboratories become 3 star, 6 laboratories were at 2 star, 11 were 1 star. Laboratory facilities respondents which thought getting adequate and timely manner mentorship were found 2.5 times more likely to get good success in the final score(AOR= 2.501, 95% CI= 1.109-4.602) than which did not get it.

**Conclusion:**

At the end of SLMTA implementation,3 laboratories score 3 star, 6 laboratories were at 2 star, 11 were at 1 star. The most important contributing factor for not scoring star in the final outcome of SLMTA were not conducting their customer satisfaction survey, poor staff motivation, and lack of regular equipment service maintenance. Mentorship, onsite and offsite coaching and training activities had shown a great improvement on laboratory quality management system in most laboratories.

## Introduction

**Figure 1:**
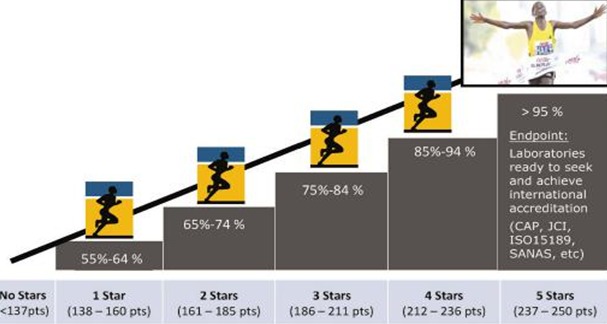
WHO/AFRO accreditation staring rate

Medical laboratory services are an essential component of health systems. Strengthening these services can combat the major infectious diseases [1]. Thus there is a need for increased direct investment in laboratory services to avoid compromising patient care [2]. This also includes quality management system to maintain and continuously improvement on the quality of laboratory processes [3]. Laboratory accreditation system is important for the acceptance of test results nationally and internationally. All medical services need reliable laboratory support for taking proper action, formulating policies and making decisions [4]. In USA study indicates that 6% to 12% of laboratory errors guide the patients at risk of inappropriate care and potentially of adverse events, and from 26% to 30% of errors have a negative impact on other aspects of patient care [5]. By recognizing this gaps of the current state of laboratories and the requirements of ISO 15189 particular requirements for quality and competence, Ethiopia has implemented the WHO-AFRO Stepwise Laboratory Quality Improvement Process, SLMTA in 2009 in order to help laboratories with stepwise recognition of evolving fulfillment of the ISO 15189 standard instead a grading system of pass-fail [6]. After the launching of WHO-AFRO Stepwise Laboratory Accreditation Process in Ethiopia 2009, from the laboratories selected for accreditation program Addis Ababa Health research and Laboratory were the only lab score four star based on WHO AFRO assessment checklist (November 2011) up from a baseline of 0-star [7]. After this the health bureau heads and officials of Addis Ababa city government take a direction to cascade this program to the health institutions (Hospitals and health centers). At base line, all the assessed laboratories were at the SLMTA zero star rating, (minimum for 1 Star: 55% of Standard), measured based on the WHO-AFRO checklist from this one could infer that there might be more problems with maintaining laboratory quality management system in the capital city of Ethiopia, Addis Ababa (Figure 1). SLMTA is a new practice and in process of development in our country. Therefore, there is a gap in knowledge and availability of data, thus an urgent need to strengthen data and to make accessible evidence based information to the professional and the community on the outcome and factor affecting SLMTA and giving useful baseline information to policy and decision makers, program managers for all efforts that will be made to improve laboratory quality in future were the corner stone of this study.

## Methods

### Study Area

This study was conducted in Addis Ababa, Ethiopia. Located at the heart of the country with the area of about 540 square kilo meters, it is the biggest city in the country and a chartered city having three layers of government namely, city government at the top, 10 sub cities in the middle and 116 woreda, hosting population of 2, 854, 462 [8]. It has 34% primary health coverage and 100% geographical health coverage, there are 6 regional, 2 NGO-supported, 30 private, 5 federal, 1 defense, 1 prison and 1police hospitals laboratories; 70 (currently functional) public and 4 NGO-supported health centers laboratories, 7 public, 500 private and 31 NGO supported clinics laboratories [9]. SLMTA were implemented on thirty one health facility laboratories (6 Hospital and 25 health centers), among them 29 facility laboratories were eligible for this study by using exclusion /inclusion criteria,4 hospital laboratory and 25 district health center laboratory which are found under city government of Addis Ababa and have been providing the clinical laboratory service for the People of the city and surrounding areas were assessed their status of the Quality System Essential before and after implementation of SLMTA at each Laboratory Facility by WHO-AFRO SLIPTA checklist.

### Study period and design

The study used an Institutional based cross sectional study design that employed a retrospective and prospective data collection approach on the participated institution of medical laboratory in SLMTA in city government of Addis Ababa. The data was collected from February-April 2014.

### Source population and study population

All health institution laboratories which are found in city government of Addis Ababa, Ethiopia were the source population of this study and the study population was all the participated institutional medical laboratories in SLMTA program on laboratory quality management system which are located in Addis Ababa city government. Inclusion and Exclusion Criteria The participated institutional medical laboratory in SLMTA program having base line and final assessment result/data was included in this study while, the institutional medical laboratory which is not participated in SLMTA, participated laboratory having insufficient data and who are not willing to participate in the study, Laboratory professional who are not at their during SLMTA & Professional who have less than one year service experience were excluded.

### Sampling Procedure

Purposive sampling technique was applied for the secondary data and the study was conducted on all the participated institutional medical laboratories in SLMTA program which full fill the Inclusion Criteria. The respondents of the questioner was selected based on the following sample size determination, the laboratory department head and the quality officer was selected by purposively and the rest was based on simple random sampling by the data collector. The sample size of the study participant for questioner respond and aiming to point out the determinate factors for the outcome of SLMTA was determined by using single population proportion formula by considering: because of the absence of previous study take p=50%, Level of significance = 0.05 Marginal of error (d) = 5%, Sample size = n Z (a/2) = Z-score at 95% confidence interval = 1.96. The formula for calculating the sample size (n) was:

n=Z_α/2_^2^P(1-P)/d^2^; n=(1.96)^2^*0.5*0.5/(0.05)^2^=384

Based on the profile of the health institution laboratory professional, there are 6 laboratories professional in the health center and 18 laboratory professional in the hospital based on BPR. In Addis Ababa there were 25 health centers and 6 government hospitals participated in SLMTA program. In each of the health centers there are six laboratories professional where as there are 18 professionals in the hospitals which account a total 108 of 258 laboratory professionals. Since the calculated sample size is greater than the total population, correction factor was done based on the finite population formula (nf), therefore the sample size was reduced to;

nf = 1+n/N, nf =154

After adding 10% for missing non response, the sample size will be 154 + 15 = 169, but because of exclusion criteria the sample, respondents become 144.

### Data collection and quality control method

The data source for the study was the results of base line & final assessment and questioner survey of the participated institution of medical laboratories on SLMTA program in Addis Ababa city government. To collect this information a standardized data extraction form which is originally developed by WHO -AFRO and adapted locally by including baseline and final data of all the 12 quality system essential and a pretested questioner was used to collect the factors associated with the outcome of SLMTA. Before utilizing these tools a pretesting was done at Minillik II Hospital and NSL woreda 06 Health Center prior to the actual data collection period. Instructions on how to use the Questioner survey was made clearly at the data collection form. The principal investigator and the supervisor were supervising closely to follow the day-to-day data collection process and ensure completeness and consistency.

### Data management and data analysis

Data was entered by using EPI-Data version 3.1. Data quality was check by the PI, supervisor and data collectors. Before doing the analysis, the entire data was cross checked for reliability and completeness on the collected hard copy data and soft copy of the entered data. Data was exported and analysis performed using SPSS (version 20). Descriptive statistics were computed. Value less than 0.05 were considered as statistically Significant. Variables with a statistically significant association (p<0.05) at univariate logistic analysis were entered and analyzed by multiple logistic regression analysis to control the confounding variables. The final result of the laboratory was conducted using the external assessors (who were not participating as the mentors), who were trained as Technical assessor by Ethiopian National Accreditation Office (ENAO), made all measurements in order to avoid biased. Based on the checklist the minimum score for the star level is 138 (55%) points of the standard, based on the final scores of 12 quality system essential elements were transformed the data in to two groups which is those laboratories which scored 138 (55%) and above as good status or star level (assigned as 1) and those laboratories which scored 137(< 55%) points and less as poor status or unable to score a star (assigned as 0) (Figure 1).

### Ethical consideration

Ethical clearance was obtained from the ethical committee of the Addis Ababa University, school of Clinical laboratory science. An official letter of cooperation was also collected from the university to the study sites and the Permission was also obtained from the AA Regional Health Bureau. The information that was collected by the study was stored in a file, without mentioning the name of the study site (institution), but a code number was assigned to it. Such information's was not be revealed to anyone except the principal investigator and was kept locked with key.

## Results

### Laboratories baseline and final assessment results

In this study, 29 health institution laboratory were participated which full fill the inclusion criteria, among them 20 were health center and 4 were hospitals. Record review was done on the baseline and final assessment of SLMTA and the reviewed data indicate that the base line score of these 29 laboratory facilities ranges from 23 (9.2%) to 85 (34%) and indicate that all 29 laboratory facilities were in zero star level. The final assessment finding indicate that there was a significant improvement in average scores, this proved to be true using paired T-test (141.4; range of 65-196, 95%CI =86.275-115.5, p = 0.000). Finally 3 laboratories become 3 star (2 health centers and 1 Hospital), 6 laboratories were at 2 star (1 hospital &5 health center), 11 were at 1 star (1 hospital, 10 Health center) and the rest were at zero star out of a possible five star (Table 1).

**Table 1:** Average Total baseline and final SLMTA result of laboratory based on the 12 LQMS, in AA, Ethiopia, 2014

Serial No.	Site code no-	Baseline Result			Final result		
		Total	Achievement	Star level	Total score	Achievement	Star
1	01	40	16%	0+	196	80.32%	3+
2	02	28	11.2%	0+	191	78.72%	3+
3	03	50	20%	0+	189	77.45%	3+
4	04	39	15.6%	0+	182	74.0%	2+
5	05	61	24.4%	0+	180	73.1%	2+
6	06	39	15.6%	0+	170	69.1%	2+
7	07	63	25.2%	0+	168	68.2%	2+
8	09	31	12.4%	0+	163	66.2%	2+
9	10	30	12%	0+	161	65.8%	2+
10	11	39	15.6%	0+	159	65.16%	1+
11	12	36	14.4%	0+	158	64.75%	1+
12	14	31	12.4%	0+	154	63%	1+
13	15	37	14.8%	0+	154	63%	1+
14	16	43	17.2%	0+	150	61.4%	1+
15	17	43	17.2%	0+	149	61%	1+
16	18	36	14.4%	0+	144	59%	1+
17	19	40	16%	0+	141	57.7%	1+
18	20	40	16%	0+	141	57.7%	1+
19	21	28	11.2%	0+	138	55%	1+
20	22	44	17.6%	0+	138	55%	1+
21	23	56	22.4%	0+	132	54%	0+
22	24	36	14.4%	0+	124	49.9%	0+
23	25	44	17.6%	0+	117	46.8%	0+
24	26	31	12.4%	0+	111	43.02%	0+
25	27	23	9.2%	0+	107	42%	0+
26	28	44	17.6%	0+	90	39.34%	0+
27	29	41	16.4%	0+	91	37.29%	0+
28	30	85	34%	0+	73	29.9%	0+
29	31	49	19.6%	0+	65	27.45%	0+

+ =star level

### Performance of laboratory on quality management system essential

**Figure 2:**
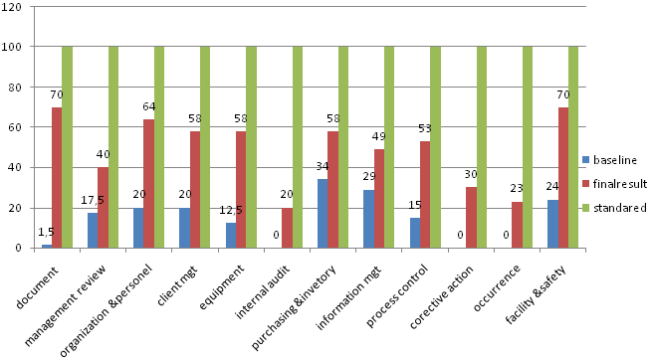
The average total assessment scored of 29 laboratories in 12 quality management systems essential in government health facilities of Addis Ababa using WHO-AFRO checklist, 2014

Based on the finding of this study after the mentorship, most district laboratories improved their scores in client management with an average of 58% from 20% of baseline result and organization and personnel achieved more than 64% scores. In management reviews, facilities and safety and occurrence management from baseline scores of 40%, 70% and 23%, respectively. Average scores for implementation of corrective actions was 30% and in case of occurrence & process control improved an average of from 0% to 23, 15%to 53% respectively. Corrective action, occurrence management and internal audits showed the highest percentage change compare to the base line results. Moreover, among the SLMTA participated health institution laboratory the highest score were achieved in document and record& facility and safety (Figure 2).

### Factors associated with the outcome of SLMTA

#### Socio demographic characteristics of the participants

In this study a total of 144 laboratory professional working in the 29 health facility were participated. About 61.1% of participants were male, the mean age of the participants were 29.21 (SD, 3.71). About 54.2% of participants were married followed by never married. One hundred four (72.2%) of them were Bsc degree in their educational status and 26.4% were Diploma. 39.6% of respondents had 6-10yrs work experience in the health facilities. When we come to the current position of the respondents in the health institution 46.5% was Laboratory bench worker and only one respondent 1(0.7%) was satisfied in his salary payment (Table 2).

**Table 2:** Socio demography characteristics of laboratory professionals who was participating in this study in Addis Ababa, Ethiopia, 2014

Variable	Frequency	Percent
**SEX**		
Male	88	61.1
female	56	38.9
**Educational status**		
Diploma	38	26.4
BSC	104	72.2
MSC	2	1.4
**professional Qualification of the respondents**		
Laboratory technician	38	26.4
Laboratory Technologist	104	72.2
Others	2	1.4
**Work experience in the current health institution**
1-2years	10	6.9
3-5years	43	29.9
6-10years	57	39.6
>10years	34	23.6
**Current position in the health institution**		
Laboratory head	29	20.1
Quality officer	28	19.4
Safety officer	20	13.9
Laboratory bench worker	67	46.5
**Salary satisfaction**		
Ye	1	0.7
No	143	99.3

#### The reason for not fully implement LQMS in their laboratory

In this study there is a chance to evaluate the reason behind that the laboratory professional for not exercising the laboratory quality system essentials in their laboratory. Based on the finding, 76% of the respondents disclosed that their facilities have no work plan and budget for laboratory specific purpose and lack of resources accounts 24% which is followed by absence of system in the health system. 105(73.4%) of the participant respond that there is no enough equipment in their laboratory and 115 (79.9%) of the lab equipment did not serviced according to the scheduled in the laboratory because of poor resource allocation and 53.9% the available equipment don't conduct preventive maintenance in the laboratory. According to the participants’ response, 91.7% replayed that their laboratory lay out and size was not adequate enough for laboratory operation due to the poor engineering lay out and followed by lack of knowledge and training on laboratory requirement during building construction. Due to the lack of motivation 27(18.8%) the laboratory didn't communicate regularly with upper management and 54(37.5%) of the laboratory professionals did not conduct their customer satisfaction survey because of poor staff communication and poor recourse allocation (Table 3).

**Table 3:** Factors affecting LQMS Essential in their laboratory in Addis Ababa, Ethiopia, 2014

Variables	Freq.	%
**Availability of work plan and budget for laboratory**		
Yes	34	23.6
No	110	76.4
**Communication with the upper mgt regularly**		
Yes	85	59
No	59	41
**Timely manner and adequate coaching and mentoring (n = 143)**		
Yes	80	55.9
No	63	44.1
**Customer satisfaction assessment activities**		
yes	89	62.2
no	54	37.8
**Laboratory provide uninterrupted testing service (143)**		
yes	22	15.4
no	121	84.6
**Laboratory deliver client results within the established TAT**		
yes	68	47.6
no	75	52.4
**Adequate Size and layout of the laboratory**		
yes	12	8.3
no	132	91.7

#### Association between outcome of SLMTA with different factors

This study explores the association of the outcome of SLMTA with different variables. The bivariate analysis showed that, there are a statically significant association between the outcomes of SLMTA with regular staff meeting, getting adequate training how to implement SLMTA, coaching and mentoring, assessment of customer satisfaction, availability of enough equipment, equipment routinely serviced, workload. Based on the finding of this study none of the socio demographic variables were found to be statically significant association with the outcome of SLMTA. However, the variables that were found to be significantly associated with the outcome of star level of SLMTA by the bivariate analysis were entered in to multiple logistic regression model to be regressed simultaneously. The multiple logistic regression model analysis made evident that performing customer satisfaction survey, timely and adequate mentorship, enough and routinely serviced equipment were statically significantly associated with the outcome of star level of SLMTA at P-value less than 0.05. Regarding to timely and adequate mentorship, laboratory facilities respondents which thought getting adequate and timely manner mentorship were found 2.5 times more likely to get good success in the final status of improvement project (AOR= 2.501, 95% CI= 1.109-4.602) than which did not get it and concerning to customer satisfaction survey, those laboratory which didn't perform their customer satisfaction survey were two point two six one times more likely to get less final result than laboratory which are conducting their customer satisfaction survey (AOR= 2.261, 95% CI= 1.851-6.007).

## Discussion

This study has been conducted in twenty nine district laboratories in city government of Addis Ababa with the aim of assessing the outcome of SLMTA and factors associated with it according to WHO /AFRO SLIPTA checklist. According to base line assessment result the mean baseline score were 41.7 which means at baseline, all laboratories scored zero stars on the WHO-AFRO SLIPTA star scale, which is similar with studies conducted in Burkina Faso national center for research and Training on Malaria to see the impact of SLMTA on Quality Management System and Study conducted at Lesotho to determine the improvement of the quality of testing services in public laboratories [10, 11]. When we look at the impact of training and mentorship activities that had been given in this 29 district health facilities, the finding indicate that there was a significant improvement in average scores (141.4; ranging from 65to196, p = 0.004). This is similar with studies conducted in Copenhagen on 51 units, South African, Zambia & Lesotho [12–15]. The associated factors were regular staff meeting, satisfaction with current salary, getting adequate training how to implement SLMTA, coaching and mentoring, assessment of customer satisfaction, availability of enough equipment, equipment routinely serviced, staff motivation like vaccination and is comparable with Wattanasri N et al study in Thailand [16]. Based on the reviewed data as indicated as in Figure 2 corrective action, occurrence management and internal audits showed a competency gap among laboratory professionals prior the SLMTA training and this study have also a comparable finding with abdosh [17].

105(73.4%) of the participant respond that there is no enough equipment in their laboratory and 115 (79.9%) of the lab equipment did not serviced according to the scheduled in the laboratory because of poor resource allocation and 53.9% the available equipment don't conduct preventive maintenance in the laboratory which is comparable study with study done in developing country laboratory, where services had the lack of adequate resources and necessary equipments [18] and study done by Peti CA et al state that the major determinate factors for providing quality laboratory services in developing countries were shortage of trained and skilled personnel; lack of equipment maintenance, poor supply-chain management systems, lack management commitment [19]. The current study revealed that 143 (99%) of respondents were not satisfied by their salary payment and the finding was comparable with the findings of Lyons et al, where the study found laboratory technologists were less satisfied on their job than other health professionals [20]. A study conducted in Thailand explained that factors that influenced laboratories’ readiness for quality improvement were shortage of staff, lack of knowledge, shortage of resource and poor staff commitment [21]. Which is concordant with the finding of current study which is due to the lack of motivation 27(18.8%) laboratory didn't communicate regularly with upper management and 54(37.5%) of the laboratory professionals did not conduct their customer satisfaction survey because of poor staff communication and poor recourse allocation.

### Limitation of the study

This study was conducted only in public health facility so; it does not illustrate the private health situation.

## Conclusion

At the end of SLMTA improvement project the finding indicate that there were a significant improvement in average scores (141.4; range of 65-196, 95%CI =86.275-115.5, p= 0.000) comparing from the baseline. Finally 3 laboratories become 3 star (2 health centers and 1 Hospital), 6 laboratories were at 2 star (1 hospital &5 health center), 11 were at 1 star (1 hospital, 10Health center) and the rest were at zero star out of a possible five star. The most important contributing factor for not scoring star in the final outcome of SLMTA were not conducting their customer satisfaction survey, shortage of resource, and lack of regular equipment service maintenance. According to the findings of this study mentoring, onsite and offsite coaching and training improve the laboratory quality management system
